# Genome-wide analysis of primate and rodent protein-coding and associated non-coding nucleotide sequences

**DOI:** 10.1186/1753-6561-6-S6-P9

**Published:** 2012-10-01

**Authors:** Sudhindra R Gadagkar, Luke A Rond

**Affiliations:** 1 Biomedical Sciences Program, Midwestern University, Glendale, AZ 85308, USA; 2 Arizona College of Osteopathic Medicine, Midwestern University, Glendale, AZ 85308, USA

## Background

Several mammalian species have been characterized by means of genome-wide analysis of the protein-coding sequences, but this has not been done in conjunction with the associated non-coding sequences, including regulatory regions.

## Materials and methods

We obtained gene data (coding sequences, 5' and 3' UTRs, intron sequences, and 5,000 bases of the 5' and 3' flanking regions) from Ensembl [http://www.ensembl.org] after determining the Ensembl IDs from the online database InParanoid7 [http://inparanoid.sbc.su.se] for all known orthologs among four mammalian species (two primate and two rodent): human (*Homo sapiens*), chimpanzee (*Pan troglodytes*), mouse (*Mus musculus*) and rat (*Rattus norvegicus*)*.* Evolutionary analyses were done using in-house computer programs or by means of the program MEGA-CC [1]. Homogeneity of the nucleotide substitution pattern between species was tested using the Disparity Index test [2], and selection tests were done using the *z*-test for coding sequences and Tajima's D [3] for non-coding sequences.

## Results

There was a total of 16,511 error-free sets of orthologs containing human genes, of which 7,244 were orthologous among all four species. A very small number (23, approximately 0.32%) of these four-way orthologs were determined to be undergoing adaptive evolution in the primate lineage. A majority of them (approximately 71%) were found to be evolving neutrally, with the rest (approximately 29 %) were determined to be under purifying selection. All of the 23 genes under positive selection in the primate lineage are under strong purifying selection when compared with the orthologs of both of the rodent species. On average, these genes show a lower G+C content (compared with the A+T content) in all four species, but especially in the primates. In contrast, the genes under negative or neutral selection show a high G+C content. Interestingly, while 327 genes were found to be evolving with a heterogeneous nucleotide substitution pattern between human and chimpanzee, only two of them are under positive selection, while 140 are under purifying selection, and 185 are evolving neutrally. Furthermore, as many as 21 genes are under positive selection, even though they are evolving with a homogeneous substitution pattern. We discuss these results and others, and compare them with those from the non-coding regions.

## Conclusions

Our work compares the evolution of coding sequences across four mammalian genomes (two primate and two rodent), and adds perspective to the results by means of comparisons with the associated non-coding sequences.
